# Prostaglandin E2 stimulates COX-2 expression via mitogen-activated protein kinase p38 but not ERK in human follicular dendritic cell-like cells

**DOI:** 10.1186/s12865-020-00347-y

**Published:** 2020-04-17

**Authors:** Whajung Cho, Jongseon Choe

**Affiliations:** 1 Research Center, Scripps Korea Antibody Institute, Chuncheon, Gangwon 24341 Republic of Korea; 2 BIT Medical Convergence Graduate Program and Department of Microbiology and Immunology, School of Medicine, Kangwon National University, Chuncheon, Gangwon 24341 Republic of Korea

**Keywords:** Follicular dendritic cell, Prostaglandin, Cyclooxygenase, MAPK, p38

## Abstract

**Background:**

Prostaglandin E2 (PGE_2_) is an endogenous lipid mediator of inflammation. Its production is regulated by the rate-limiting upstream enzyme cyclooxygenase-2 (COX-2). We have recently demonstrated that the major cell type expressing COX-2 in the germinal center is follicular dendritic cell (FDC). In this study, to elucidate the molecular mechanism of PGE_2_ in COX-2 production, we asked whether mitogen-activated protein kinases ERK and p38 might regulate COX-2 expression.

**Results:**

FDC-like cells were used to analyze the phosphorylation kinetics of ERK and p38 and the impact of genetic knockdown. PGE_2_ stimulation gave rise to a rapid increase of p38 but not ERK phosphorylation. In contrast, IL-1β induced phosphorylation of both MAPKs. Knockdown of p38 resulted in a marked suppression of COX-2 expression induced by either PGE_2_ or IL-1β. ERK knockdown did not significantly affect the effect of PGE_2_ and IL-1β on COX-2 induction. The differential results of p38 and ERK siRNA transfection were reproduced in the production of prostaglandins and in experiments performed with pharmacologic inhibitors.

**Conclusions:**

Our data indicate that p38 is essentially required for PGE_2_ to induce COX-2 expression in FDC-like cells. The current study helps to expand our understanding of the biological function of FDC at the molecular level and provides a potential rationale for the pharmacologic or genetic approaches to regulate p38 MAPK in the treatment of various inflammatory disorders.

## Background

Mitogen-activated protein kinases (MAPKs) are important intracellular signaling molecules responsible for the various cellular functions under normal or pathologic conditions. These serine/threonine-specific protein kinases transduce signals coming from a plethora of extracellular stimuli including cytokines and prostaglandins (PGs). There are three major families of MAPKs, ERK, p38, and JNK, and subfamilies in each type [1]. ERK and p38, in particular, appear to be critical molecules based on the results obtained from knockout (KO) animals. Mice with single KO of certain subfamily of either ERK or p38 undergo embryonic death. For example, *Erk2* KO mice and *p38α* KO mice are embryonic lethal [2, 3] while the single KO of any *Jnk* gene does not lead to the embryonic lethality [4]. These earlier studies underscore the physiological importance of ERK and p38 MAPKs.

Follicular dendritic cells (FDCs) are peculiar stromal cells observed in the B cell follicles of peripheral lymphoid organs [5]. Their cellular origin of mesenchymal stromal cells is a distinction of FDCs from other cellular components in the secondary lymphoid tissues most of which derive from hematopoietic stem cells [6]. The biological roles of FDC include B cell recruitment to the follicles, presentation of native antigens on the surface, and provision of survival, proliferation, and differentiation signals to germinal center (GC) B cells [7–10]. In the course of efforts to understand the GC reactions at the molecular level, we have recently suggested another interacting pathway between FDC and B cells. We demonstrated the expression of cyclooxygenase-2 (COX-2) molecule in FDC-like cells in vitro and further verified FDCs as the major cell type expressing COX-2 in situ [11]. COX-2 is a well-known enzyme induced by various factors including inflammatory stimuli and serves the rate-determining role in the production of PGE_2_ [12]. Using the experimental system containing FDC-like cells, we showed that PGs promote the survival of GC B cells by preventing apoptosis [13], augment the antigen-presenting ability of B cells by increasing CD86 expression [14, 15], and exert a positive feedback effect on COX-2 expression [16]. These in vitro results and our previous results with COX-2 KO mouse imply the important role of COX-2 molecule in the inflammation taking place in the immune tissues [17].

We have previously observed that ERK and p38 MAPKs are involved in COX-2 expression in FDC-like cells. For example, LPS-induced COX-2 expression was inhibited by ERK and p38 inhibitors, which was verified by the actual induction of phosphorylation of these MAPKs by LPS [18]. TGF-β-stimulated COX-2 induction also required ERK and p38 [19]. In the present study, we extended our previous reports and explored the intracellular pathway of PGE_2_-induced COX-2 expression in FDC-like cells. PGE_2_ treatment resulted in a rapid increase of p38 but not ERK phosphorylation. In contrast, IL-1β, whose effect was compared in parallel with PGE_2_, induced phosphorylation of both MAPKs. Knockdown of these MAPKs revealed that p38 is essential for PGE_2_ to induce COX-2 expression in FDC-like cells, in line with the phosphorylation results. Our data provide a potential rationale for the pharmacologic or genetic approaches to regulate p38 MAPK in the treatment of various inflammatory disorders.

## Results

**Fig. 1:**
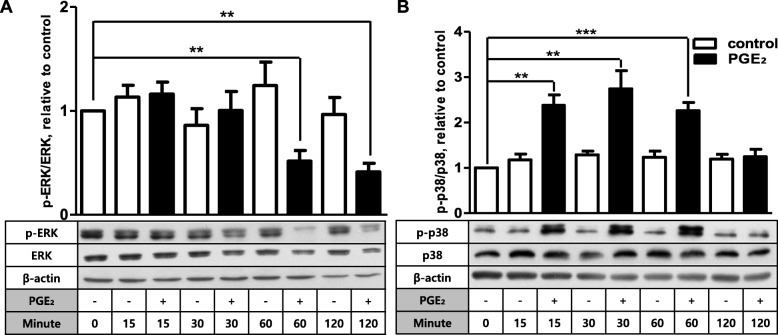
PGE_2_ selectively increases phosphorylation of p38 in FDC-like cells. The total and phosphorylated levels of ERK (**a**) and p38 (**b**) MAPKs were examined by immunoblotting before and at the indicated time points after culture of FDC-like cells in the presence or absence of PGE_2_ (1 μM). β-Actin was used to show equal loading of cell lysates. Representative immunoblots and statistical analysis data (mean ± SEM) of three independent experiments are shown. An asterisk(s) indicates a significant difference (**, *p* < 0.01; ***, *p* < 0.001)

**Fig. 2:**
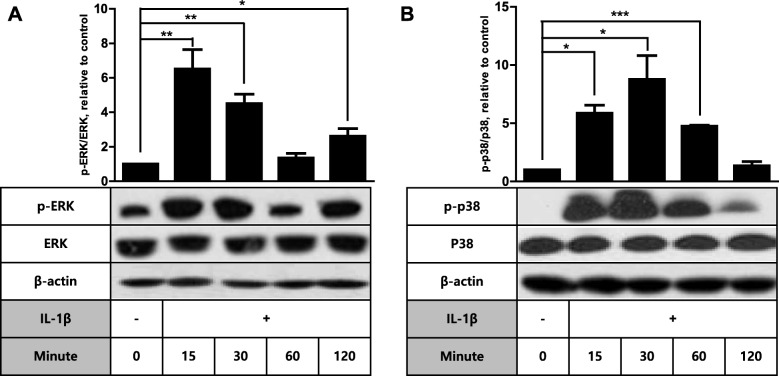
IL-1β induces phosphorylation of both ERK and p38 in FDC-like cells. The total and phosphorylated levels of ERK (**a**) and p38 (**b**) MAPKs were examined by immunoblotting before and at the indicated time points after culture of FDC-like cells in the presence of IL-1β (10 pg/ml). Representative immunoblots and statistical analysis data (mean ± SEM) of three (**a**) or two (**b**) independent experiments are depicted. An asterisk(s) indicates a significant difference (*, *p* < 0.05; **, *p* < 0.01; ***, *p* < 0.001)

We have recently demonstrated that PGE_2_ stimulates COX-2 expression in human FDC-like cells via EP2 and EP4 surface receptors on the cell surface [16, 20]. In this study, we further investigated the underlying intracellular mechanism by examining the potential role of ERK and p38 MAPKs in this process. Our earlier results suggest that both ERK and p38 molecules are involved in the signaling pathway to COX-2 expression [19]. First, the effects of PGE_2_ on the phosphorylation degrees of ERK and p38 proteins were analyzed by immunoblotting. The signaling molecule would display increased levels of phosphorylation since ERK and p38 are phosphorylated by MAP kinase/ERK kinase (MEK) to act on the target molecules [21]. PGE_2_ did not increase phosphorylated forms of ERK but rather reduced ERK phosphorylation at 60 and 120 min post-stimulation by approximately 50% compared to the control maintained without PGE_2_ (Fig. 1a). In contrast, p38 phosphorylation increase was evident from 15 min and continued until 60 min. For instance, more than 2-fold increase of p38 phosphorylation was observed at 30 min compared to the vehicle control. The elevated levels of p38 phosphorylation returned to background levels at 120 min. The enhancing effect on p38 phosphorylation was triggered by PGE_2_ because such an activation was not observed in control cultures carried out collaterally in the absence of PGE_2_ (Fig. 1b). To explore whether the differential phosphorylation induction is specific to PGE_2_, we performed the phosphorylation kinetics of ERK and p38 after stimulation with IL-1β. IL-1β is a strong inducer of COX-2 in FDC-like cells [20]. Different from PGE_2_, IL-1β treatment resulted in increased phosphorylation of both ERK and p38. For example, phosphorylation levels of both proteins were approximately 6-fold higher at 15 min after IL-1β stimulation (Fig. 2). Both PGE_2_ and IL-1β did not significantly modulate the total protein levels of ERK and p38 molecules. Taken together, these results imply that ERK and p38 molecules are differentially involved in the COX-2 induction pathways by PGE_2_ and IL-1β.

**Fig. 3:**
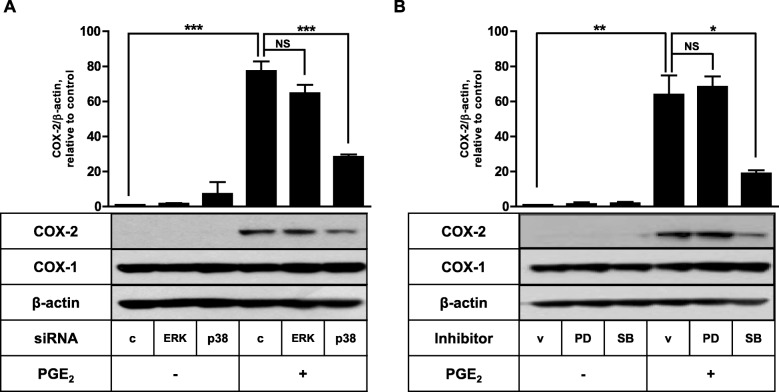
p38 MAPK is necessary for the PGE_2_-induced COX-2 expression in FDC-like cells. **a** The effects of ERK and p38 knockdown on COX-2 induction by PGE_2_ were examined with FDC-like cells that had been transfected with control or siRNA against ERK or p38 before the addition of PGE_2_ (1 μM). **b** The effects of PD098059 (PD, 50 μM) and SB203580 (SB, 10 μM) on COX-2 induction by PGE_2_ were examined. FDC-like cells (1 × 10^5^ cells) were cultured in the presence of PD098059 or SB203580 for 30 min and then added with PGE_2_ (1 μM) for 8 h. The expression levels of COX-1, COX-2, and β-actin were measured by immunoblotting. Representative immunoblots and statistical analysis data (mean ± SEM) of three independent experiments are shown. An asterisk(s) indicates a significant difference (*, *p* < 0.05; **, *p* < 0.01; ***, *p* < 0.001; NS, non-significant). v, vehicle

**Fig. 4:**
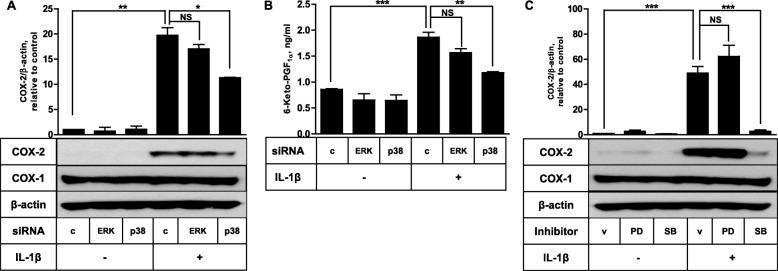
IL-1β-stimulated COX-2 expression in FDC-like cells depends on p38 MAPK. **a** The effects of ERK and p38 knockdown on COX-2 induction by IL-1β were examined with FDC-like cells that had been transfected with control or siRNA against ERK or p38 before the addition of IL-1β (10 pg/ml). The expression levels of COX-1, COX-2, and β-actin were measured by immunoblotting. **b** The amounts of 6-keto-PGF_1α_ in culture supernatants of *(A)* experiments were measured by EIA as described in *Methods*. **c** The effects of PD098059 (PD, 50 μM) and SB203580 (SB, 10 μM) on COX-2 induction by IL-1β were examined. Representative immunoblots and statistical analysis data (mean ± SEM) of three independent experiments are shown. An asterisk(s) indicates a significant difference (*, *p* < 0.05; **, *p* < 0.01; ***, *p* < 0.001; NS, non-significant). v, vehicle

To determine the role of ERK and p38 in PGE_2_- and IL-1β-triggered COX-2 expression, the impact of ERK and p38 protein knockdown was analyzed. As shown in Fig. 3a, PGE_2_-induced COX-2 expression was significantly inhibited by p38 knockdown. About 65% reduced COX-2 protein was obtained in the cells transfected with p38 siRNA. Administration of FDC-like cells with siRNA against ERK did not significantly modulate the COX-2 induction. Similar results were obtained in experiments performed with pharmacologic inhibitors of ERK and p38 (Fig. 3b). PGE_2_-induced COX-2 expression was significantly inhibited by SB203580 but not by PD098059. SB203580 is an inhibitor of p38 and PD098059 is a specific inhibitor of MEK-1, an upstream kinase of ERK. These inhibitors did not affect the expression of COX-1. COX-2 induction by other PGs, beraprost and PGF_2α_, was affected similarly by p38 knockdown (data not shown). Next, we conducted the siRNA transfection experiment to investigate the effects of ERK and p38 protein knockdown in IL-1β-induced COX-2 expression. As presented in Fig. 4, IL-1β-induced COX-2 expression was suppressed approximately 50% by p38 knockdown and almost completely by SB203580. ERK knockdown or PD098059 treatment did not affect the COX-2 induction stimulated by IL-1β. The modulation of COX-2 protein expression either by IL-1β stimulation or p38 siRNA inhibition was reflected in the actual production of prostaglandins (Fig. 4b).

## Discussion

**Fig. 5:**
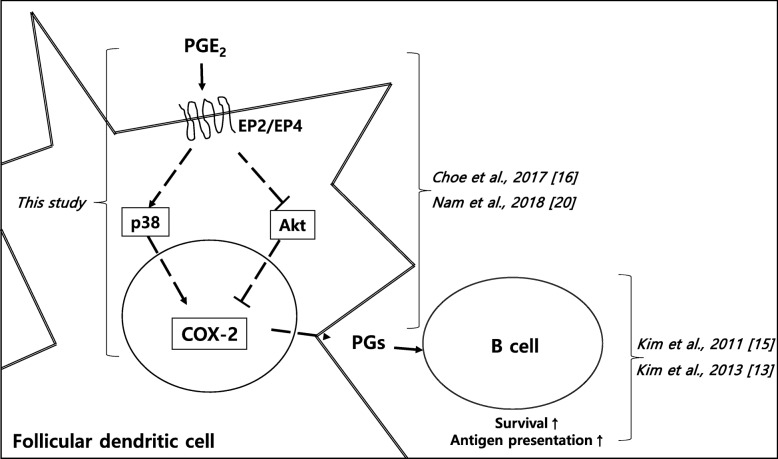
Proposed model for the central role of p38 mitogen-activated protein kinase in COX-2 expression in human FDC. The paracrine or autocrine effects of PGE_2_ are exerted to FDC via EP2 and EP4 receptors. PGE_2_ promotes COX-2 expression by stimulating the activity of p38 activity and repressing that of Akt. PGs released from FDC take part in the GC reactions in a paracrine fashion to regulate important functions of lymphocytes. For example, PGE_2_ enhances the survival and antigen-presenting capability of GC B cells

Using primary human FDC-like cells, we demonstrate here the essential requirement for p38 MAPK in COX-2 protein expression instigated by PGE_2_. PGE_2_ selectively induced phosphorylation of p38, and p38 knockdown gave rise to a marked suppression of PGE_2_-induced COX-2 expression. The current data suggest another intracellular mechanism on the positive effect of PGE_2_ on COX-2 expression. We have previously reported that PGE_2_ promotes COX-2 expression in FDC-like cells by inhibiting Akt phosphorylation [16]. Therefore, PGE_2_ that might be produced by autocrine or paracrine fashion in the GC appears to bind to EP2 or EP4 receptors on the surface of FDC [20] and enhances COX-2 expression by activating p38 MAPK and by suppressing Akt activity simultaneously. The upregulation of p38 phosphorylation and downregulation of Akt phosphorylation occurred with comparable kinetics in FDC-like cells. The elevated COX-2 expression and resultant PG production may contribute to the GC reactions in part by promoting the survival and antigen-presenting capability of B cells [13–15]. Figure 5 shows our working model for COX-2 induction mechanisms in FDC that is based upon our experimental results presented here and previously.

p38 MAPK was required while ERK was dispensable for COX-2 induction in FDC-like cells. Compared with siRNA knockdown that did not deplete all the p38 proteins in target cells, almost complete inhibition of COX-2 was obtained with p38 inhibitor treatment, implying the essential requirement of p38 MAPK. Similar to this result, many investigators report that p38 MAPK plays the essential role in the IL-1β-stimulated COX-2 expression in human lung fibroblasts and MDA-MB-231 breast cancer cells [22, 23]. However, unlike FDC-like cells, not only p38 but also ERK mediates COX-2 induction by IL-1β in human colorectal cancer cells [24]. In a sharp contrast, IL-1β-treated COX-2 induction in human endometrial stromal cells was inhibited by ERK but not p38 inhibitors [25]. These data imply that COX-2 expression is regulated by distinct MAPKs in different cell types. In our hands, MDA-MB-231 breast epithelial cells did not respond to IL-1β, and COX-2 expression was not modulated as reported elsewhere [26].

Both PGE_2_ and IL-1β displayed the common feature of p38 activation and p38 requirement for COX-2 induction. This result may be regarded as a molecular mechanism for our recent observation of a synergistic effect of PGE_2_ and IL-1β in COX-2 induction [20], implying that the synergy of PGE_2_ and IL-1β may be achieved partly by synergistic augmentation of p38 activity. Inasmuch as FDC is localized in the microenvironment of GC, the synergy suggests that PGE_2_ takes part in the early stage of inflammatory response by providing more PGs to the ongoing responses initially triggered by pro-inflammatory cytokines such as IL-1β. IL-1β is produced at the initial stage of inflammation [27]. This speculation for the inflammation-amplifying role for PGE_2_ is supported by several reports from other research groups. In response to endogenous PGE_2_, pro-inflammmatory cytokines are released from monocytes and synovial fibroblasts [28, 29].

Although the physiological relevance of the current data is unclear, the results are in line with the general concept that MAPK p38 is involved in inflammation while ERK plays pivotal roles in cellular proliferation [30]. PGE_2_ did not modulate the proliferation of FDC-like cells in the range of 0.01~10 μM concentrations (Supplementary Fig. 1). In this context, the increased ERK phosphorylation after IL-1β treatment is enigmatic since this cytokine does not significantly affect the growth of FDC-like cells. The target molecules and biological function of IL-1β-induced phosphorylated ERK remain to be investigated.

## Conclusions

In conclusion, we demonstrate in this study the requirement of p38 MAPK in PGE_2_-stimulated COX-2 protein in FDC-like cells. Considering the pivotal role of PGE_2_ in the inflammatory microenvironment, p38 MAPK regulation via pharmacologic or genetic approaches may be taken in the treatment of various inflammatory disorders.

## Methods

### Antibodies and other reagents

Antibodies against ERK, phosphorylated ERK, p38, phosphorylated p38 MAPKs were purchased from Santa Cruz Biotechnology, Inc. Specific antibodies to COX-1 and COX-2 were purchased from Cell Signaling Technology. Anti-β-actin antibody was obtained from Sigma-Aldrich. Secondary antibodies were horseradish peroxidase (HRP)-conjugated anti-mouse IgG (Southern Biotech) and anti-rabbit IgG (Koma Biotech). PGE_2_ was purchased from Cayman Chemical, and IL-1β from R&D Systems. All the siRNA duplexes were obtained from Ambion Inc. as described previously [19]. PD098059 and SB203580 were purchased from A.G. Scientific, Inc.

### Cell culture

FDC-like cells are primary cultured cells. They are fibroblastic adherent cells obtained through the enzyme digestion of human tonsils as described previously [31]. The purity of cells was more than 98% when assessed by a FACSCalibur (BD Biosciences) for the expression of FDC-specific marker 3C8 as shown previously in the supplementary figure of representative phenotypic analysis data [32]. They proliferate under the conventional culture condition and display antigenic and functional features of FDC [33]. FDC-like cells are used until they exhibit degenerate features in culture. Cell culture media was RPMI-1640 media (GIBCO) containing 10% fetal bovine serum (Hyclone), 2 mM L-glutamine, 100 U/ml penicillin G (Sigma-Aldrich), and 100 μg/ml streptomycin (Life Technologies).

### Immunoblotting

Cultured FDC-like cells were lysed in a buffer containing 50 mM Tris-HCl (pH 7.5), 150 mM NaCl, 0.5% Nonidet P-40, 1 mM dithiothreitol, 5 mM EDTA, and protease inhibitor mixture at 4 °C. For clarification, cell lysates were centrifuged at 12,000 rpm/min at 4 °C for 10 min. The supernatants were harvested, and then total protein concentrations were measured using Bio-Rad protein assay reagent. Cellular proteins were heated at 95 °C for 3 min and then separated by SDS-PAGE, followed by electrotransfer to polyvinylidene difluoride (PVDF) membranes. PVDF membrane was then blocked for 1 h with Tris-buffered saline containing 0.05% (v/v) Tween 20 and 5% (w/v) non-fat dry milk. The membranes were incubated with appropriate antibodies and then with SuperSignal West Pico Chemiluminescent Substrate (Pierce) and exposed to X-ray films.

### siRNA transfection

FDC-like cells were cultured to 50 ~ 60% confluence in 100 mm plates. For each plate, 40 nM of each siRNA and 24 μl lipofectamine (Invitrogen) were separately diluted in 400 μl serum-free medium without antibiotics and then mixed together. The mixture was incubated at RT for 45 min. The plates containing FDC-like cells were washed with serum-free medium, added with 5 ml serum-free medium, and then with the mixture. The plates were incubated at 37 °C for 8 h, followed by the addition of a growth medium containing 10% serum. Cells were used for the experiments after 48 h of additional incubation. The degree of gene-silencing was verified by immunoblotting.

### Enzyme immunoassay (EIA) to measure PGs

The amounts of PGI_2_ (6-keto-PGF_1α_) in the culture supernatants of FDC-like cells were measured using EIA kits conforming to the manufacturer’s instructions (Cayman Chemical).

### Statistical analysis

Statistical analyses of data were carried out using GraphPad Prism 5.04 software to be presented as the mean ± SEM of three independent experiments. The statistical significance of differences was determined using Student’s *t* test. *P* < 0.05 was considered significant.

## Supplementary information

**Additional file 1: Supplementary Figure 1.** PGE_2_ does not modulate the proliferation of FDC-like cells. The effect of PGE_2_ on cell growth was examined by culturing FDC-like cells in the presence or absence of indicated concentrations of PGE_2_ for 72 h. Indomethacin (Indo) was used at 100 μM after determining its inhibitory concentration. The impact on cell proliferation was measured by Cell counting kit-8 (Dojindo Molecular Technologies) according to the manufacturer’s instructions. Representative results and statistical analysis data (mean ± SEM) are shown. An asterisk indicates a significant difference (*, *p* < 0.05).

## Data Availability

The dataset of the current study is available from the corresponding author at a reasonable request.

## Abbreviations

COX: Cyclooxygenase
FDC: Follicular dendritic cell
GC: Germinal center
MAPK: Mitogen-activated protein kinase
MEK: MAP kinase/ERK kinase
NO: Knockout
PG: Prostaglandin
