# Supplementary figures and images for: Prostaglandin E2 stimulates COX-2 expression via mitogen-activated protein kinase p38 but not ERK in human follicular dendritic cell-like cells
Source: BMC Immunol. 2020 Apr 17;21:20. doi: 10.1186/s12865-020-00347-y (PMC7165408; doi:10.1186/s12865-020-00347-y)

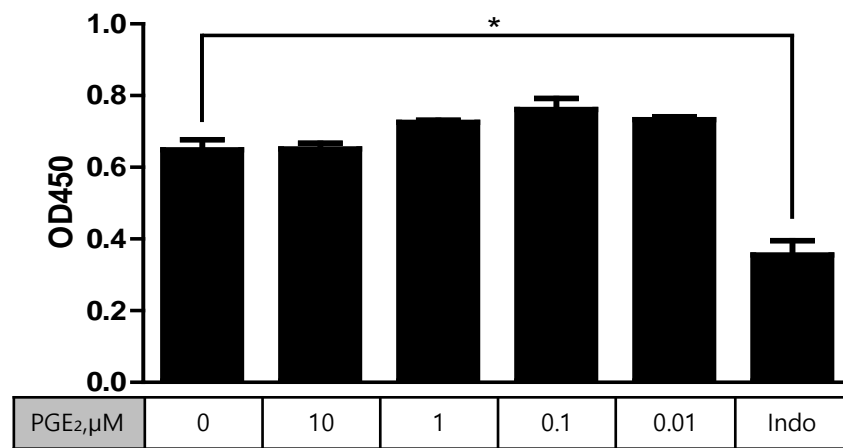

Supplementary Figure 1

Supplement: Supplementary file 1 — Additional file 1: Supplementary Figure 1. PGE2 does not modulate the proliferation of FDC-like cells. The effect of PGE2 on cell growth was examined by culturing FDC-like cells in the presence or absence of indicated concentrations of PGE2 for 72 h. Indomethacin (Indo) was used at 100 μM after determining its inhibitory concentration. The impact on cell proliferation was measured by Cell counting kit-8 (Dojindo Molecular Technologies) according to the manufacturer’s instructions. Representative results and statistical analysis data (mean ± SEM) are shown. An asterisk indicates a significant difference (*, p < 0.05). [file 12865_2020_347_MOESM1_ESM.pdf]
